# Behçet’s Disease Presenting With Massive Haemoptysis Due to Pulmonary Artery Aneurysms: A Case Report

**DOI:** 10.7759/cureus.115636

**Published:** 2026-09-02

**Authors:** Khalid Elgorashi, Takudzwa J Dhlandhlara, Asad Khan, Arvind Sinha, Rahul Mukherjee

**Affiliations:** 1 Internal Medicine, University Hospitals Birmingham NHS Foundation Trust, Birmingham, GBR; 2 Internal Medicine, Heartlands Hospital, Birmingham, GBR; 3 Rheumatology, University Hospitals Birmingham NHS Foundation Trust, Birmingham, GBR; 4 Respiratory Medicine, Heartlands Hospital, Birmingham, GBR

**Keywords:** behçet’s disease, haematemesis mimic, massive haemoptysis, pulmonary artery aneurysm, vasculitis

## Abstract

An information technology worker of South Asian heritage in his 30s presented with haematemesis and central chest pain. Initial evaluation, including oesophagogastroduodenoscopy, was unremarkable. Subsequent imaging revealed multiple pulmonary artery aneurysms. Blood tests showed a negative autoimmune profile and raised inflammatory markers. Infectious, thromboembolic and connective-tissue differentials were excluded. A history of oral ulcers with genital ulceration, together with vascular involvement, fulfilled International Criteria for Behçet's Disease (ICBD). Given the life-threatening risk, we initiated intravenous methylprednisolone followed by cyclophosphamide, later transitioning to azathioprine with a structured steroid taper. Haemoptysis ceased promptly, and follow-up imaging showed aneurysm resolution. This case illustrates how haemoptysis can masquerade as gastrointestinal bleeding and emphasises the need to consider pulmonary artery aneurysms in young patients with compatible systemic features, where early imaging and rapid immunosuppression may be lifesaving.

## Introduction

Behçet's disease (BD) is a chronic relapsing multisystem inflammatory disorder characterised by recurrent oral aphthous ulcers, genital ulceration, ocular inflammation, skin lesions, and variable involvement of the vascular, gastrointestinal, and nervous systems. Although considered a rare disease in most Western countries, prevalence varies markedly according to geography, ranging from less than 1 per 100,000 population in Northern Europe and North America to more than 100 per 100,000 in countries along the historic Silk Road, including Türkiye, the Middle East, and East Asia [1,2]. The disease typically presents in young adults between the second and fourth decades of life and can result in substantial morbidity and premature mortality when major organ involvement occurs [1].

The pathogenesis of BD remains incompletely understood but is thought to arise from an interaction between genetic susceptibility and environmental triggers. The strongest genetic association is with the human leukocyte antigen (HLA)-B51 allele, which confers a significantly increased risk of disease development and is present in a substantial proportion of affected patients [2,3]. In genetically predisposed individuals, exposure to infectious or other environmental antigens may trigger dysregulated innate and adaptive immune responses characterised by neutrophil hyperactivity, T-helper 1 and T-helper 17 cell activation, endothelial inflammation, and cytokine excess. The resultant vasculitis is unique in its ability to affect arteries and veins of all sizes, distinguishing BD from many other systemic vasculitides [1-3].

Vascular involvement develops in approximately 25-40% of patients and is a major determinant of long-term outcome [1,4]. While venous thrombosis is the most common vascular manifestation, pulmonary artery aneurysms (PAAs) represent one of the most severe and feared complications of the disease [1,4,5]. PAAs occur in fewer than 5% of patients with BD but account for a disproportionate share of disease-related mortality because rupture into the bronchial tree can result in catastrophic haemoptysis [1,5]. Historically, mortality rates approached 50% in untreated patients, with pulmonary arterial involvement recognised as one of the strongest predictors of death [5,6].

The diagnosis of pulmonary artery involvement can be challenging because haemoptysis may be misinterpreted as haematemesis, particularly when respiratory symptoms are subtle or absent. Careful history taking is therefore essential. Features favouring haemoptysis include coughing preceding blood expectoration, frothy bright-red blood, and associated pleuritic chest pain, whereas haematemesis is more commonly associated with nausea, abdominal symptoms, or coffee-ground vomitus [7]. Modern computed tomography pulmonary angiography (CTPA) has become the imaging modality of choice for detecting PAAs, defining their extent, and monitoring treatment response [8].

Rapid suppression of vascular inflammation is central to management. Current European Alliance of Associations for Rheumatology (EULAR) recommendations advocate high-dose glucocorticoids combined with cyclophosphamide as first-line therapy for life-threatening vascular BD, including PAAs [4,5]. This approach aims to prevent aneurysm expansion and rupture by controlling the underlying vasculitis rather than treating the aneurysm as an isolated structural lesion. Increasing evidence also supports the use of tumour necrosis factor (TNF)-α inhibitors, such as infliximab and adalimumab, in refractory, relapsing, or severe cases, reflecting a growing shift towards targeted biologic therapy [9,10].

We present a case of BD in a young man whose PAAs initially masqueraded as gastrointestinal bleeding. The case highlights the diagnostic challenges associated with differentiating haemoptysis from haematemesis and illustrates the importance of early recognition and prompt immunosuppressive treatment in preventing potentially fatal outcomes.

## Case presentation

Clinical history

An information technology worker of South Asian heritage in his 30s, previously healthy, presented to the accident and emergency department with haematemesis associated with central chest pain. Upon presentation, he had no melena, heartburn, or abdominal pain. He was an ex-smoker. He did not drink alcohol and had not been on anticoagulants or non-steroidal anti-inflammatory drugs (NSAIDs). He had no other comorbidities and was not on any long-term medications.

Physical examination was unremarkable, and his haemoglobin was 126 g/L. The Glasgow-Blatchford score was 1, and he was discharged with a course of high-dose omeprazole. He re-presented the following day with coffee-ground vomiting and left-sided chest pain. He was haemodynamically stable and was admitted for further work-up. He had an oesophagogastroduodenoscopy (OGD), which was grossly unremarkable.

During admission, he reported episodes of producing "frothy blood-streaked sputum" after coughing. These features were suggestive of haemoptysis rather than haematemesis. Other supporting features for haemoptysis were the absence of associated abdominal pain; rather, he had pleuritic chest pain. These clinical features raised suspicion for parenchymal or vascular pulmonary pathology rather than gastrointestinal bleeding. He mentioned that he had had recurrent self-limiting painful oral ulcers lasting up to three weeks at a time on at least six occasions in the previous two years. He reported two previous episodes in his late 20s of painful and scarring penile ulcers, which self-resolved after two weeks on each occasion. There was no history of scrotal ulcers, ocular symptoms, skin lesions, arthritis, or neurological symptoms. There was no family history of autoimmune or inflammatory conditions.

Chest CT (Figure 1) showed peribronchial lesions in the right lung, including a single lesion in the middle lobe measuring 29 × 22 mm in the axial plane and three lesions in the lower lobe measuring 18 × 15 mm, 19 × 15 mm, and 16 × 13 mm in the axial plane, respectively. The laterally placed lesion in the right lower lobe demonstrated surrounding ground-glass, which was assumed to reflect haemorrhage. There were no thrombi seen within the aneurysms.

**Figure 1:**
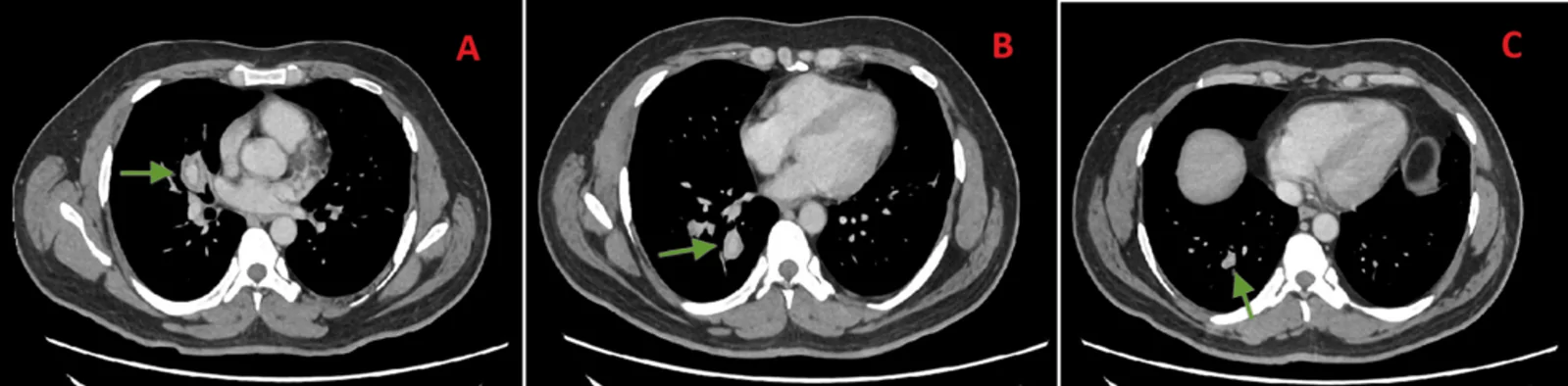
CT thorax axial section images at presentation showing peribronchial lesions (green arrows) in the right lung, suggestive of aneurysms in the right main pulmonary artery (A) and the bronchial artery branches of the right middle lobe (B) and right lower lobe (C).

Echocardiogram showed no features of pulmonary arterial hypertension (PAH), no valvular pathology and an ejection fraction of 57% ± 5%. Laboratory investigations are highlighted in Table 1. Of note, serum free light chain levels and IgG and IgA levels were elevated at presentation, indicating polyclonal B-cell activation driven by pro-inflammatory cytokines.

**Table 1:** Laboratory investigations at presentation and at four weeks after initiation of treatment. Hb: haemoglobin; WCC: white cell count; ESR: erythrocyte sedimentation rate; CRP: C-reactive protein; ANA: anti-nuclear antibody; ANCA: anti-neutrophil cytoplasmic antibody; IGRA: interferon gamma release assay; HLA: human leucocyte antigen; TB: tuberculosis; PCR: polymerase chain reaction; HBsAg: hepatitis B surface antigen; MC&S: microscopy, culture, and sensitivity; Ab: antibody

Test name	Reference range	At presentation	Four weeks after initiating treatment
Haematology			
Hb (g/L)	130-171	122	120
WCC (x10^9^/L)	3.26-11.20	11.60	13.36
Platelet Count (x10^9^/L)	150-400	475	280
ESR (mm/hr)	2-10	73	5
Biochemistry			
Sodium (mmol/L)	133-146	133	139
Potassium (mmol/L)	3.5-5.3	4.1	3.7
Urea (mmol/L)	2.5-7.8	3.4	6.2
Creatinine (mmol/L)	64-104	79	74
CRP (mg/L)	<4	78	<1
Immunology			
Serum Free Kappa (mg/L)	3.30-19.40	113.59	-
Serum Free Lambda (mg/L)	5.70-26.30	60.63	-
Kappa/Lambda Ratio	0.26-1.65	1.87	-
IgG (g/L)	6.00-16.00	39.15	-
IgA (g/L)	0.80-2.80	10.00	-
IgM (g/L)	0.50-1.90	0.43	-
C3 (g/L)	0.75-1.65	1.62	-
C4 (g/L)	0.14-0.54	0.25	-
ANA	-	Positive; Titre 1/50	-
ANCA	-	Not detected	-
Quantiferon IGRA	-	Negative	-
HLA B51	-	Negative	-
Microbiology			
Sputum MC&S	-	Negative	-
Sputum TB PCR	-	Negative	-
HIV 1 and 2 Serology	-	Negative	-
Syphilis Serology	-	Negative	-
HBsAg	-	Negative	-
Hepatitis C Ab	-	Negative	-

Differential diagnosis

Behçet's Disease (BD)

Further exploration of clinical history was key, as our patient reported having previous recurrent painful oral ulcers and scarring genital ulcers. In addition, imaging evidence of PAA denoted vascular involvement. Using the International Criteria for Behçet's Disease (ICBD) scoring system [11], he had 2 points for oral aphthosis, 2 points for genital aphthosis, and 1 point for vascular manifestations, giving a total of 5 points (at least 4 points are required for diagnosis). Pathergy testing was not performed because of the urgency to initiate high-dose immunosuppressive therapy; while not required for diagnosis here, it could have further substantiated the clinical impression [1,8].

Hughes-Stovin Syndrome (HSS)

This rare disorder, characterised by the combination of multiple PAAs and venous thrombosis, is often considered an incomplete variant of BD. HSS lacks the recurrent oral and genital ulceration typical of BD and is defined primarily by vascular features [12]. In our patient, there were no clinical features of venous thrombosis.

Takayasu Arteritis

This was considered due to the patient’s age and the large-vessel nature of the PAAs, but there was no history of limb claudication, no bruits on examination, and CT angiography demonstrated a normal aorta and major branches, with aneurysms confined to the pulmonary arteries. This made Takayasu arteritis highly unlikely [13].

Post-tubercular Mycotic Aneurysms

PAAs are a known possible complication of tuberculosis (TB) [14]. Our patient had no history or current clinical features of TB. Microbiology analysis and imaging had ruled out the possibility of TB.

Mycotic Aneurysms

Bacteremia is a cause of inflammation of blood vessels with subsequent thinning of intimal layers and aneurysm formation [15]. There was no evidence of active infection in our patient.

Syphilis

Tertiary syphilis is well documented to cause vascular complications and aneurysms mainly involving the aorta [16]. Syphilis serology was negative, and our patient had no features of tertiary syphilis.

PAH or Collagen Vascular Disorders

PAH and connective tissue diseases can lead to vascular abnormalities [17]. Transthoracic echocardiography (TTE) showed no features of PAH.

Treatment

The patient was initially given intravenous (IV) pulse methylprednisolone 500 mg daily for three days, followed by cyclophosphamide infusion 1000 mg 14 days later. Following IV methylprednisolone pulse therapy, the patient was transitioned to oral prednisolone 60 mg daily for two weeks, which was subsequently tapered by 5 mg every two weeks. The cyclophosphamide regimen consisted of two further induction doses every two weeks, followed by seven maintenance doses every three weeks. Additionally, the patient was prescribed cotrimoxazole 960 mg three times a week for *Pneumocystis jirovecii* pneumonia prophylaxis, omeprazole 40 mg once daily for gastric protection, and mesna for bladder protection. While on cyclophosphamide, the patient had regular surveillance for bone marrow suppression, infections, haematuria, liver function and kidney function. No adverse events were recorded.

Outcome and follow-up

The patient reported significant clinical improvement after two weeks of starting immunosuppressive treatment. At the four-week follow-up, he reported no recurrence of haemoptysis or cough, and no new oral or genital ulcers. Examination revealed no active mucocutaneous lesions, no signs of ocular inflammation, and normal cardiorespiratory findings. Laboratory tests showed normalisation of inflammatory markers (Table 1).

By the eight-week review, he remained clinically stable. Repeat CT pulmonary angiography (Figure 2) showed resolution of the right main pulmonary artery and bronchial branch artery aneurysms. Ground-glass opacities seen on the initial scan, consistent with alveolar haemorrhage, had resolved. No new aneurysms or thrombotic lesions were identified.

**Figure 2:**
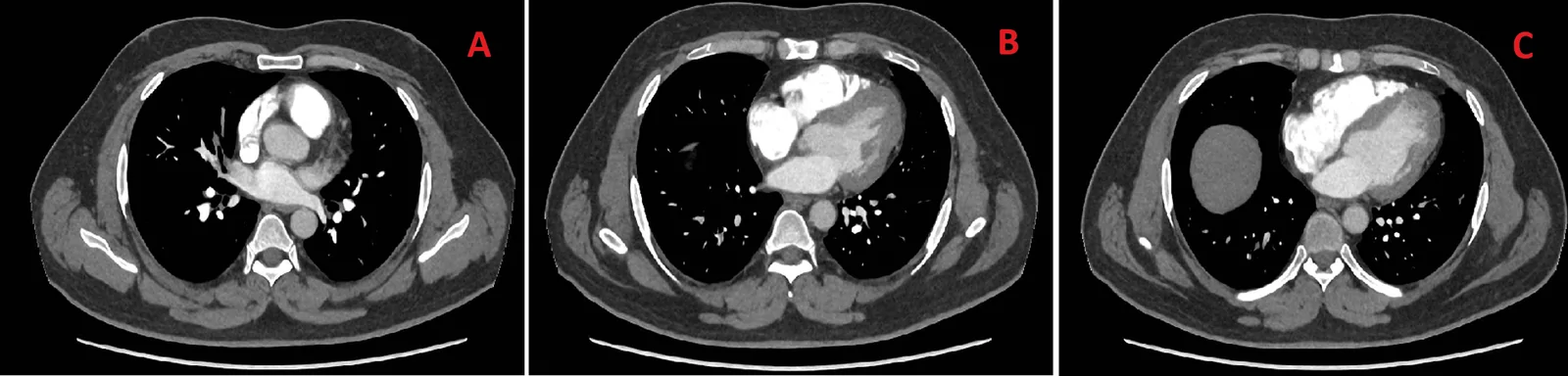
CT thorax axial section images at eight weeks after initiation of treatment showing resolution of the aneurysms in the right main pulmonary artery (A) and the bronchial artery branches of the right middle lobe (B) and right lower lobe (C).

At three months, he was started on azathioprine, which he tolerated well. The steroid dose had been tapered further without relapse, and six months into follow-up, repeat chest imaging confirmed maintained resolution of aneurysms and no new vascular involvement (Figure 3). Pulmonary function tests remained normal. The multidisciplinary team agreed to maintain azathioprine and complete steroid taper, with consideration of anti-TNF therapy if there was evidence of disease relapse or aneurysm progression in future. Plans were made for 6-12 monthly imaging and ongoing joint rheumatology-respiratory monitoring.

**Figure 3:**
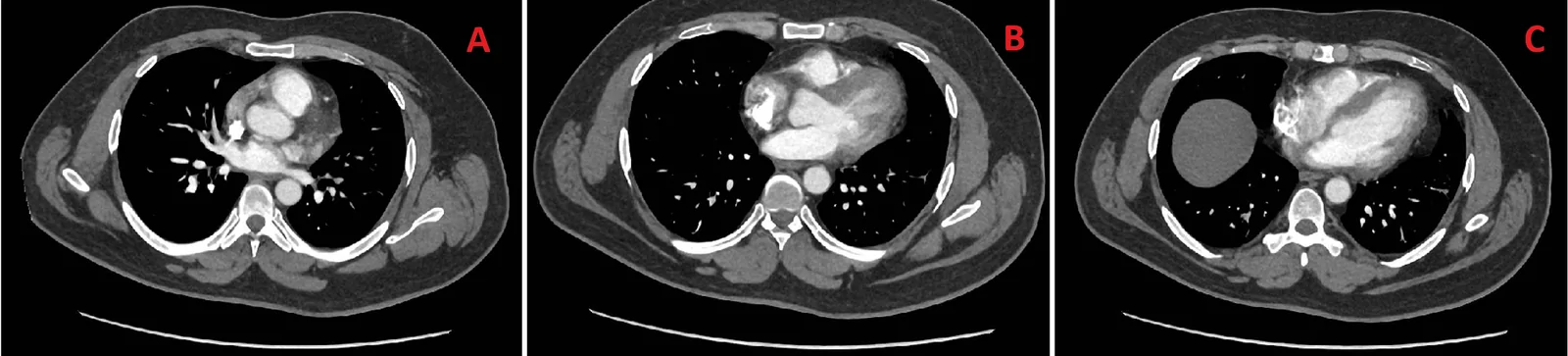
CT thorax axial section images at six months after initiation of treatment showing maintained resolution of the aneurysms in the right main pulmonary artery (A) and the bronchial artery branches of the right middle lobe (B) and right lower lobe (C).

## Discussion

This case highlighted that differentiating haematemesis from haemoptysis in the acute setting might not be straightforward. A detailed history is key, and features such as associated chest pain or abdominal symptoms may help to differentiate the two. Often haemoptysis can mimic gastrointestinal bleeding and potentially lead to misdiagnosis [7].

Cross-sectional imaging is invaluable in investigating this overlap, especially to rule out serious and potentially fatal causes of haemoptysis. PAAs, though rare, should be considered in patients with recurrent haemoptysis and systemic symptoms. PAAs may rupture directly into the bronchial tree, producing large-volume haemoptysis without preceding respiratory symptoms.

High-dose corticosteroids combined with cyclophosphamide have traditionally been the first-line induction regimen for life-threatening vascular BD, including PAAs, due to their rapid onset of action and availability in acute care settings [1]. In this case, the decision to initiate cyclophosphamide was driven by the severity of presentation, the need for immediate disease control, and the absence of prior immunosuppressive therapy. However, emerging evidence supports the use of TNF inhibitors as a potent alternative, particularly in refractory cases. Hamuryudan et al. demonstrated that patients with pulmonary artery involvement treated with anti-TNF agents such as infliximab or adalimumab achieved faster remission and greater radiologic improvement compared to those receiving cyclophosphamide. This has been further consolidated by the New England Journal of Medicine, which highlighted early use of biologics in severe cases with acceptable safety profiles when infection risk is appropriately managed [9]. Alibaz-Öner and Direskeneli further emphasised the role of biologics not only in refractory disease but also as potential early-line therapy in severe vascular involvement, aligning with a broader shift towards targeted treatment [10].

Current management of vascular BD is supported by the EULAR recommendations, which advocate rapid suppression of vascular inflammation to prevent irreversible organ damage and fatal complications. For PAAs, EULAR recommends high-dose glucocorticoids combined with cyclophosphamide as first-line induction therapy, while monoclonal anti-TNF agents are recommended for refractory, relapsing, or severe disease. These recommendations provide a structured framework for treatment escalation, progressing from conventional immunosuppressive therapy to targeted biologic agents when adequate disease control is not achieved [4,5]. In our patient, the excellent clinical and radiological response to corticosteroids and cyclophosphamide was consistent with this treatment paradigm.

PAAs are one of the most serious manifestations of BD and remain a major determinant of disease-related mortality. Massive haemoptysis resulting from aneurysmal rupture into the bronchial tree is the principal mechanism of death. Historical series reported very poor outcomes, with mortality rates approaching 50% and two-year survival rates of approximately 60% in untreated or inadequately treated patients. The advent of earlier diagnosis, aggressive immunosuppressive therapy and multidisciplinary management has substantially improved outcomes; however, pulmonary arterial involvement continues to be associated with significant morbidity and mortality [5,6].

Although immunosuppression remains the cornerstone of treatment, interventional and surgical approaches may be required when medical therapy fails, when aneurysms enlarge despite treatment, or when life-threatening haemoptysis occurs. Several case series and reviews have demonstrated favourable short-term outcomes with endovascular techniques, including coil embolisation, vascular plugs, covered stents, and glue embolisation, which are increasingly preferred because of lower procedural morbidity compared with surgery. Surgical options such as aneurysmorrhaphy, lobectomy, pneumonectomy, or pulmonary artery ligation are generally reserved for selected patients with localised disease, giant aneurysms, or failed endovascular therapy. Nevertheless, surgery remains technically challenging because ongoing vasculitic inflammation may predispose to postoperative complications and recurrence [18,19].

As a single-case observation, the findings cannot be generalised to the wider BD population. Histopathological confirmation of pulmonary arterial vasculitis was not obtained because of the high procedural risk, and the diagnosis was based on clinical criteria and characteristic radiological findings. Pathergy testing was not performed due to the need for urgent treatment, and the relatively short follow-up period limits assessment of long-term relapse risk and durability of the observed radiological response.

## Conclusions

Haematemesis with associated chest pain should raise suspicion of large-volume haemoptysis. Cross-sectional imaging should be considered when OGD findings are inconclusive. Multidisciplinary collaboration is essential in diagnosing and managing complex systemic diseases.
